# The loss of dignity: social experience and coping of women with obstetric fistula, in Northwest Ethiopia

**DOI:** 10.1186/s12905-019-0781-7

**Published:** 2019-07-01

**Authors:** Debrework Tesgera Bashah, Abebaw Gebeyehu Worku, Mezgebu Yitayal, Telake Azale

**Affiliations:** 1 School of Nursing College of Medicine and Health Sciences, University of Gondar, P.O. Box 196, Gondar, Ethiopia; 2 Department of Reproductive Health Institute of Public Health, College of Medicine and Health Sciences University of Gondar, Gondar, Ethiopia; 3 Amhara National Regional State Health Bureau, Bahir Dar, Ethiopia; 4 Department of Health Service Management and Health Economics Institute of Public Health, College of Medicine and Health Sciences University of Gondar, Gondar, Ethiopia; 5 Department of Health Education and Behavioral Sciences Institute of Public Health, College of Medicine and Health Sciences University of Gondar, Gondar, Ethiopia

**Keywords:** Coping mechanisms, Loss of dignity, Obstetric fistula, Social experience,Women

## Abstract

**Background:**

Obstetric fistula is a debilitating condition resulted from poorly (un) managed prolonged obstructed labor. It has significant psychosocial and economic consequences on those affected and their families. Data regarding experiences and coping mechanisms of Ethiopian women with fistula is scarce.

**Methods:**

Qualitative design was employed with in depth interview technique by using open ended interview guide. Eleven fistula patients waiting for surgical repair at the fistula treatment center of Gondar Specialized Referral Hospital were selected with typical case selection. Thedata were audio-taped, transcribed and translated from Amharic to English. Open code version 4.03was used to organize data and identify themes for analysis.

**Results:**

The age of participants of the study ranged between 19 to 43 years. Ten of them were from rural areas. Regarding their educational status eight cannot read and write. Similar number were either separated or divorced. Six of them lived with obstetric fistula without treatment from one to five years. Five women related their condition to their fate. The women faced challenges in role performance, marital and social relationships and economic capability. Frequent bathing, use of stripes of old clothes as a pad, self-isolation and hiding from being observed, wearing extra clothes as cover, increasing water intake and reducing hot drinks and fluids other than water were the ways they have devised to cope with the incontinence.

**Conclusion:**

The study participants reported that they experienced deep sense of loss, diminished self-worth and multiple social challenges. They coped with the incontinence in various ways among which some were non effective and might have continuing negative impact on woman’s quality of life even after corrective surgery. Developing bridging intervention for early identification and referral could reduce period of women’s suffering.

**Electronic supplementary material:**

The online version of this article (10.1186/s12905-019-0781-7) contains supplementary material, which is available to authorized users.

## Background

Obstetric fistula (OF) refers to an abnormal connection between epithelial wall of the reproductive tract and the bladder or/and the rectum predominantly caused by unattended obstructed labour [1]. Commonly it occurs when there is cephalo pelvic disproportion, a sustained pressure from the baby’s presenting part deprives blood flow to the surrounding tissues of the mother’s pelvis then prolonged ischemia will cause tissue necrosis leading to fistula formation [2]. Obstetric fistula is one of the severe birth injuries occurring almost exclusively in the developing world mainly sub-Saharan countries, where access to quality obstetric care is lacking [3, 4]. It is estimated that two million women have an obstetric fistula with more than 50,000 new cases occur annually [5].

In Ethiopia, it is estimated that there are about 39,000 women suffering from untreated fistula and 3700 women who develop fistula injuries each year, causing life-long disabilities and poor quality of life [6, 7]. In Ethiopia Maternal health service utilization is very low. The 2011 Ethiopian Demographic and Health Survey (EDHS) reported that among 34% of the women attended ANC visits; only 11.7% used skilled delivery attendants. Therefore, poor compliance to seek for skilled delivery attendant in turn perpetuates risk for the occurrence of delivery complications including obstetric fistula [8].

In developing countries women lack the power to decide on their reproductive right, pregnancy timing and place of delivery. Most women with fistula reported that their husbands or mothers-in-law make the decision even when they should seek health care services [5, 9]. Birth injuries including obstetric fistula are resulted from multiple delays during labor and delivery.

Prolonged and obstructed labor often results in maternal mortality, and those who survive mostly lose their baby and are left with an obstetric fistula [10]. Obstetric fistula is the formation of an abnormal opening between vagina and surrounding structures: the bladder or the rectum. Consequently it leaves a woman with constant leak of urine or feces or both [11]. It also has significant psychosocial and economic consequences on those affected and their families [12, 13]. Social isolation and financial challenges due to obstetric fistula are beyond the medical conditions [14, 15]. The condition is linked with social exclusion and more devastating to the woman’s social status [16]. The continuous leakage of urine makes the affected women prone to stigmatization and subject to social humiliation [17, 18].

Care and treatment seeking of women with obstetric fistula is influenced by community perception about the condition [19, 20]. Where there is misperception about the cause the affected women do not disclose their condition for fear of being blamed. Therefore, they hide themselves from being identified and continue to suffer silently from psychosocial and physical consequences [10, 21]. Reports showed that there are high rates of divorce and abandonment and the women experienced the fistula as a direct assault on their ability to fulfill their social expectations [13, 15, 22, 23]. The care and support given to women depends upon community understanding. The coping mechanisms also differed among women [10]. Though there are reports on lived experiences and quality of life of women with repaired fistula [24–27], voices of Ethiopian women regarding their social experience before receiving corrective surgery and their coping strategies are sparse in the literature. Moreover, information on how women are suffering from fistula cope and knowledge of real life experience would help to plan feasible prospects after treatment to maintain their identity as a woman.

This study address the gap by presenting the social experiences before treatment and their coping mechanisms. Documenting the difficulties of women with obstetric fistula in their own voices deepens insight into the nature of the problem and serves as a call to action to develop bridging intervention for early identification and treatment in order to reduce period of their suffering from obstetric fistula.

## Methods

### Study setting

The study was conducted at Fistula Treatment Center of the University of Gondar Specialized Referral and Teaching Hospital from September to November 2017.University of Gondar Specialized Referral and Teaching hospital provides fistula treatment service at the new fistula treatment center established in 2011 in collaboration with Women and Health Aliance International (WAHAI) with the support of the United Nation Population Fund (UNFPA) and the Fistula Foundation. The center has 70 beds established with the aim of treating 100 women with fistula each month and aimed to be an international training centre of excellence for surgeons from Ethiopia and abroad. Patients come to the center from different areas around either by hearing information from previously treated women in their village or through campaigns for identifying women with incontinence. The women may come accompanied by their intimate familymember or with professional, who identified their condition. In this center patients are not expected to pay for the fistula care they receive, rather the center covers to and fro transport fee for both the woman and her accompany. Once the patients are screened and diagnosed to have fistula they will be admitted in the ward to receive care.

### Study design and sampling

Since one can learn about a woman’s health through compassionate and effective listening of her talk about all her own feeling, pain, suffering and any joy as part of her experience [28], qualitative case study approach was used to explore fistula patients’ experience before treatment and their coping mechanisms. Participants were women who had developed obstetric fistula and waiting for repair surgery at the Fistula treatment center of the University of Gondar Specialized Referral and Teaching Hospital. Interviews were conducted with women after admission to the center for corrective surgery. Eleven women were enrolled in the interview purposively. Two nurses working in the center has reviewed patients’ medical record and recruited women for interview.Data saturation and information need determined the sample size.

### Data collection

A convenient time for an interview was arranged; the first author and one trained female nurse collected the data through face-to-face interview in Amharic language, the nationallanguage of Ethiopia. Open ended interview guide allowing women to tell their experience since they contracted the problem was employed. The guide consisted of the following discussion points like 1.How did the women experienced the problem (how was fistula happened to you?how did you recognize that you have fistula?) 2. What social challenges did they faced while they were living with the problem (how do you explain your relationship with your husband, family and neighbors? What were you doing for your livelihood? 3. How did they cope with challenges(how did you coped with the continuous leak? What were you doing to overcome the social challenges?) 4. How did they find the treatment service? (How did you know that your problem is treatable? Who did accompany you to the treatment center?)(Additional file 1). Probing was used to clearly understand meanings of experiences and there were flexibility in order of questions. The informant was the major speaker. The interview lasted between 45 min to 1: 05 h.

### Data analysis

Open code version 4.03(Department of Epidemiology and Global Health, University of Umea)was used to organize themes for data analysis. The audio-taped data were transcribed, compared for consistency with the notes taken during interview and then translated from Amharic to English. The investigators andthe independent translator ascertained the quality of translation. All the responses of the interviewees were written on word document and saved as open code text.Transcripts were read thoroughly and meaning units were extracted and condensed without losing quality and meaning. Words, phrases, or statements that raised related issues were identified as meaning units andassigned similar codes under first synthesis. Those codes were aggregated and categorized to provide broad themes.Onset of fistula and perceived cause, role and relationship, experience in income source, coping with incontinence and awareness about and use of service were identified from the organized data and discussed.

## Results

### Respondents’profile

The age of interviewees ranged from 19 to 43 years. Ten of them were from rural areas. Eight were unable to read and write and three attended primary education. Currently three of the participants were living with their husband and the rest were either separated or divorced. The period women lived with the condition without treatment ranged between three months to nine years and two months with the average duration of 46.55 months (3.9 years). Among the respondents four had at least one living child and only two infants survived from the delivery that led to fistula (Table 1). The study participants, who had assisted delivery at health facility, were the ones who came to the center relatively early.Two were previously repaired for recto-vaginal fistula.

**Table 1:** Back ground information of respondents’; Social experience and coping mechanisms of women with obstetric fistula, northwest Ethiopia, Gondar

Age in range	Current Marital Status		No of children		Fetal outcome		Years with Fistula		
	Married	Divorced/separated	≥1	0	alive	stillbirth	< 1 yr	1 yr–5 yr	> 5 yr
18–25	3	1	3	1	1	3	2	2	0
26–30	0	4	2	2	1	3	0	4	0
31–35	0	2	1	1	0	2	0	0	2
> 35	0	1	2	0	0	1	0	0	1

### I. Social experiences

#### Onset and perceived cause

Respondents reported that they had labored for about 43–63 h either at home, hoping that they will give their first birth in their mother’s house as the custom encourages or on the way to health facility when the attendant assured failure of advancement of the presenting part. Five women were assisted by their neighbor, family or village’s labor attendant. Three were taken to health facility and underwent assisted or operative delivery.

*“Before I went to my mother’s house for delivery,one day morning I felt pushing down and back pain (may be labor). I had that symptom from morning to the following day evening waiting until his [husbands] grandmother comes. She was known in her village for her “skill” in helping laboring woman. When she arrived I was tired and unable to push more. She started to push my abdomen down to help baby come out. She did for several times in between she introduced her hands in but nothing was changed”. (Age 26-30 years, divorced, has no living child).*

A woman labored for two solid days stated that skilled care was sought after labor has complicated and the woman became weaker.

“*As the baby was not delivered and I was getting weaker, she talked something, they discussed among themselves. After a while people came and took me to health center.”(Age 18–25 years,married, has one living child).*

Days after delivery they experienced either of the following: feeling wetness all the time, soaked clothes when getting up from bed and failure to control urine gradually. Initially women were expecting that as if he incontinence will resolve after some time when their body recovers.

*“It happened in my first pregnancy, after Ihad labored for two days they took me to health institution, there they (health professionals) inserted an object in to my body to assist birthing and dead baby was born. After some days I had periodic spills that I thought it will improve through time. But it was getting worse from day today.”(Age 19-25 years, married, has alive child).*

In the first incident of leak most of the respondents were not aware of what had happened to them and when the leak became continuous, none of them spoke their condition to anyone thinking that the problem was specificto them. However, others observed/identified from their acts that they had a kind of ‘disease’. Before they reach the hospital and informed by health professionals most of them had not known and identify prolonged labor as a cause rather they attributed the condition either to their destiny, evil spirit or curse.

“*…Initially I was feeling that I am the only cursed woman with such problem; therefore I have not told to any one, I always cry, hated myself, and the day I was born. I was broken. But when I see other women with similar problem here (in hospital) I got friends and am slightly consoled.”* Another woman responded that “*You cannot be against to what has been intended for you at birth; it is all about my fate” (Age 31–35 years, divorced, has no child).*

### Experience in role and relation ship

Women tend to avoid doing demanding household chores to lessen the amount of leak. They don’t want to get involved in outdoor activities such as going to market to sell or buy products and fetching water. The family members and sometimes close neighbors were doing for the woman for a period of time. But later as time goes they get bored, recognize her condition as ‘incurable’ and support become diminishing. Also spouse’s family might initiate the issue of separation/ divorce.

*“At the beginning my family and neighbors were sympathetic, were advising and helping me. Through time when the problem persisted they started to be distant from me. They do not want to touch thing that I used, my seat and place became separate I am not allowed to sit other than my seat I think that they lost hope at me no one cares about me, I was just living in my mom’s house striving to hide my condition, soaked and washing all the time” (Age 31–35 years, divorced, has one child, lived with fistula for six years).*

The incontinence, coupled with inability to fulfill roles as a woman was a great challenge.Women become rejected and divorced due to the condition that left them with continuous leak and loss of newborn.

*“…There he (spouse) heard that the baby was not alive then he was not interested to take me back home and I returned to my mother’s house until I recover. On his visit my mother told him my condition and asked him to take me to health center, afterwards he never came back to visit me again…”(Age 26-30 years, separated, lived with fistula for two years and eleven months).*

Though it is challenging they became dependent on their natal family for their livelihood.

“*Four months after he recognized my problem he divorced me. I left my home there and went to my sister’s house found in small town Tselemt. I told her all what happened to me and we planned to work together sharing role, me to make tella and work in house activities and she will sell it (serve) to the customers. In this way we were leading life, later after a year I found difficulty to manage the pain and leak which goes by itself, my body has peeled off then I tend to reduce the activities and becoming dependent on her. It was unpleasant for her to continue with me in such condition since the income is very small. From day to day she complained and insults me that I smell and defaced her house, I detested my fate and left her house and moved to my grandmother’s house in another village…. She (her grandmother) was happy for my going there because she had no child living with her. When she knew my illness she felt miserable and gave me her old cloth. She shared me all what she had and also encouraged me to search for solution.”(Age 26–30, divorced, lived with fistula for three years).*

Mostly where the urinary leak is heavy, having sexual activity is found embarrassing and painful to them thus they would abstain from sexual activity. As a result some became separated or get divorced and lost all what they had in their married life.

“*Since the problem I became disabled I cannot assist him in farm work and unable to make household chores independently as I did before. They (husband’s family) started discussing on me…that their son lacks care and I am no more purposeful as a wife*.” *(Age 26-30 years, Divorced woman, has no child, lived with the condition for four years).*

Conversely those who lived with fistula for less time and younger age women reported that their spouses and relatives were supportive and accompanied them to health facility.

*“…He married me at my young age (17 years). We have one baby, the problem happened on my first delivery I did not knowsuch problem before. I suffered a lot from labor. Thanks to the doctor he saved my baby while I was left with another ‘disease’…..Now he(husband)brought me here to get treatment. He is caring for the baby at home with my mother.”(Age 18–25 years, Married woman, has one child, lived with the condition for five months).*

The women also experience difficulty in getting sleep and bored of changing beddings.

“*You cannot sleep comfortably until morning, all the bedding become soaked I have to wake up two to three times in the night to change especially when I was with my husband I worry much to make him unaware of heavy leak”(Age 18–25 years,Married woman, lived with the condition for one year and 9 months).*

Women were unable to satisfy their roles as a woman and were endured all the pain to preserve their marriage.

*“I was incapable to do heavy work which requires vigor. This problem made me weak. I spend a lot of my time washing my clothes and self. I used to cook food and clean the house with all the difficulties to save my marriage, but lately I become unable to do so and he become offended and divorced me”(Age 26–30 years,divorced,lived with the condition forthree years and two months).*

### Experience with income source

Most women had no income generating activity before fistula and were dependent on marital life.Divorce would force them to work on difficult tasks for minimum wage.

*"As I left my home I was working on (AbaTesfhager’s’* 1 *) (a neighbor of her natal family) farm for weeding. He gives me cereal (barley) and sometimes I used to sell that to get money (birr) for my expenses”.(Age 19–25 years, divorced, lived with condition for five years).*

Those who had income generating activity before fistula had stopped due to their condition.

*“Before the problem I was selling ‘tella’ [local drink], also continued after my illness but when my condition became recognized….no one was coming to my house….now I stopped it. Whenhe (husband) left me….I went to my mother’shometo live with….”(Age 26–30 years, divorced, lived with condition for four years).*

Some other women were working for very minimal payment that would not require strict hygiene.

*"I was roasting maize/barley for a woman who prepares “tella” for sell every week. She will give me on the day I complete the work, I will use that for soap, underwear and sometimes for salt and coffee.(Age 26–30 years, separated,lived with condition for two years and11 months).*

### Coping with the incontinence

Respondents reported that they were leading distressing lifefeeling sad and prefer to die as a result of their inability to keep clean or being seen as unclean.

“*It is miserable, distressing life and I prefer dying rather than living in such painful life…. Ehh (breaths deeply) my condition is the worst. I was repaired for the first fistula which happened during my first delivery. The problem recurred after two years. I have no good time since this problem. Just crying, hating my being. I came on appointment, for this relapse I was repaired twice and the urine flow became worsen I lost hope and was tried to kill myself (commit suicide).”(Age 31–35 years, Divorced, lived with for seven years).*

Three were always putting on unsighty clothes. Women who experienced both Recto-vaginal and vesico-vaginal fistulae were hiding themselves from everyone and opt to die.

*“I do not want to stand around or sit aside any body. You see I would have to kept all my suffering inside, other than my family if someone in my village had known my condition;It was very shameful for meand it was better to die rather than being in that condition.” (Age 18–25 years, divorced, has no child, lived with condition for five years).*

In trying to keep clean during the day women wore non colored clothes (that absorb and does not show the stains) or wrap colorless/ black outfits over their clothes to cover the wetting.

*“Iwore ugly ‘dirty’ clothes that does not expose the sign of urine and mystify my condition” (Age 26–30 years, divorced, lived with condition for three years).*

Another woman also added*.*

*“…mostly I won’t go out, if I should, I wear a black dress or wrap my waist with another sheet….” (Age 18–25 years, married, lived with condition for three months).*

They also use pieces of old clothes as an absorbent pad. They attempted to wash and dry “pads”in private. But in summer they could not get hidden site to dry and it also needsmore time to dry. They would not change frequently as they wish. Therefore, use smoke as a means for drying.

“*The urine pours without stopping, I use piece of clothes as a pad to protect my cloth from getting wet easily. I have to wash and change it repeatedly unless the smell is so humiliating, I will soak all the pieces at day time and wash when they sleep, the problem is you could not get private place to dry all the pieces mainly in rainy season it will not dry easily. So I will hang up it over“Tunjit”(a kind of herb with good smell) smoke.”(Age 26–30 years, divorced, lived with condition three years and two months).*

At home when they are alone they often leave the pad and let it go, but due to the skin irritation by the urine they use it intermittently.

“*At home, when the piece of cloth in my pants irritates me I will remove and let the urine flow, but this also cause itching and sores on my thigh*.” *(Age18–25 years,lived with condition for five months).*

Women explained that mostly they avoid going to visit friends, invitations and attending any social gatherings or choose particular time to go tochurch and invitation place not to be identified. But if they would do so, they had to be sure of having enough protection and in funerals of close relatives where absence could lead to social exclusion they will carry extra clothing for frequent changing or use piece of old cloth as an absorbent pad.

*“Since this ‘disease’,I go to church too early and return before people come together……..the smell is so humiliating, thus I do not want to sit with people in a closed area (where there is no free air movement). Because though I feel clean, those who know my condition pretend to cover their nose ….”(Age 18–25 years,lived with condition for one year and 9 months).*

*“It is disgraceful disease which turns a clean woman to insanitary. When I go for invitations like ‘mahiber’ [a kind of religious social gathering, ceremony], I putpieces of old cloth in my pants for adequate protection until I return.”(Age 26–30 years, divorced, lived with condition for four years).*

Another respondent added;

*“In funerals, where absence leads to family exclusion I will put on another cloth to cover the stain”(Age 26–30 years, lived with condition for two years and eleven months).*

In their day to day life,the women either restrict themselves from fluid intake to limit the flow of urine; or take more water to dilute the strong odor and reduce skin irritation.

*“After two years of the problem I decided not to take fluid as I should. Especially I avoided hot drinks like coffee to minimize the flow but I the change was minimal. Rather the urine burned my thighs again I started to take more water….I reduce water intake when I have outdoor activities”(Age 43 years,divorced, lived with the problem for more than nine years).*

### Awareness and use of service

Respondents reported that they were unaware of fistula treatment availability and perceive the problem was exceptional to them. Subsequently, they would not disclose the condition and seek care. Women heard about repair services from different sources; from a relative or community members who had known of previously repaired woman, health facility and campaigns.

*“I have never seen or heard of such disease before, and did not think it could be treatable so I take care not to be identified in the community, after years I am bored of my condition and went to health center to know the case in order to decide on my life. However they assured me that the problem is treatable and gave me a letter to go to Gondar [the place where the Fistula Treatment Center is found].” (Age 26–30 years, divorced, lived with condition for three years).*

*“I was applying green paste on my thigh, that local healer has given me to relief pain and heal the sores. It was very helpful……I met my old friend coming from town (Gondar)….I discussed all my problem with her thinking that if incase she can help me in money to pay for….she told me that government is treating women of similar problem and gave me few birrand her contact to call her when I go there… therefore I am encouraged and decided to come herefor getting relief”(Age 31–35 years, divorced,lived with the condition for six years).*

After they became aware of repair services, though some were reported to have supportive husband, another were challenged of how to reach there (due to their condition), to get travel cost and accompanying person. They were also challenged of lack of family support who takes over their roles at home.

*“Though I have heard of service availability I could not go as soon as I then my baby was only seven months. What will be fed to such baby? To whom do I could leave my child to care for at home? I do not have mother or sister. So I had to stay until he (the baby) able to feed other than breast milk…” (Age 18–25 years, married, lived with the condition for one year and nine months.*

“*…the woman (our neighbor working in kebele) has taken me to Woreda(district) women’s affairs office; we heard that the Ambulance service is free for people like me. She sent me to health center. Again they sent me here [*treatment center]*. If I had not got theses service how would I reach here? I may remain there until I get sufficient money or would have dead. Here, they helped me a lot, offered me a bed and food. No problem of water for bathing and washing.I saw the doctors [most people coming to hospitalcall every health professional ‘doctor’] arevery caring”*.(*Age 26–30 years, divorced,lived with condition for 4 years).*

Among the participantwomen, three had sought care from health facility at least once. Two had got operation for recto vaginal fistula in Addis Ababa Hamlin Fistula Repair Center, and waiting repair for the urinary incontinence. One had undergone two unsuccessful surgical repairs.

*“In my attempt to commit suicide my uncle who was living in Addis has heard of my condition and took me to hospital there,I found women with similar problem. I was stayed there for two months, got treatment and becameable to control the feces (bowel symptom) but not the urine flow. They advised me to return to Bahir Dar or Addis Ababa another time for the urine leak. In between I heard that Gondar has been giving the service and I came here” (Age 31–35 years, divorced, 7 years with condition).*

## Discussion

This study has explored the social experiences and coping mechanismsof 11 Ethiopian women were living with obstetric fistula. The women experienced uncontrolled leak as gradual incident during their postnatal periods and were unaware of what was happening in their body. Initially some hope that it will resolve after some time when their body recovers. Many did not identified prolonged labor as a cause, rather attributed it to either their fate or being cursed. This is supported by previous findingsin which women do not know the actual cause of fistula and relate it to reward for wrong deed in youth ages [29] supernatural causes [27, 30, 31] evil doing of her rival (husband’sanother wife) [32], and an intervention by skilled attendant [32]. The knowledge on actual cause and nature of obstetric fistula is still low and calls for intervention.

Women reported that they had general body pain, weakness and constant leak which formed sores due to friction and has limited them from doing routine chores. Different studies from Tanzania and Uganda also reported experience of altered role performance due to genital sores, exhaustion and inability to walk normally [20, 29].

Besides these difficulties many women suffered loss of dignity as a woman and adverse marital status change continued over time and seven of them were divorced and one separated since they recognized that they have this problem.Disrupted marital relationships due to fistula were reported in a number of studies [10, 20, 29, 30, 32, 33].Only three were living in their marital home with all the difficulties, similarly other studies reported that divorce is not always the case [34, 35]. In this study women shared that their social relationship was challenged and experienced social isolation. This is similar to previous studies in which women in Nigeria who experienced ostracism in all direction; from husbands, families and communities [29]. Findings show that this isolation will also continue after treatment [27, 34, 36] thus, being incontinent and challenges to maintain hygiene coupled with alienation from family and friends affects their self-confidence.

None of respondents had own income source activities or engaged in activities other thancooking or preparing edible products and get less income. Reports confirm that women with fistula lost their jobs [32, 37] and unable to contribute to family earnings [10] and rarely employable after treatment [34]. It is important to note that obstetric fistula has led women to lead life of dependency. They were unable to generate income due to various reasons. As the condition made them weak they faced physical challenges to work with their family on income sources. At times they were working on non-gainful jobs that donot require strict cleanness like weeding and roasting as a laborer to at least pay for hygiene supply and assist family. Respondents worry much about the leak, various losses and lack of support. Especiallyas the problem persists support becomes diminishing in all aspect resulting in hopelessness.

Our finding showed that fistula has challenged women’s life style and the coping ability and strategies were dependent on source of support, and community’s view/understanding. To cope with the physical signs of incontinence most women used homemade absorbent pads commonly made of old cloths as reported in previous studies [10, 32]. Though women had a difficulty to get necessary hygiene supplies they keep bathing and washing of used stripe (pads)often with wateronly and hang on herb smoke to mask the odor. Womenin Ghana use scented soap for washing and spray perfumes whenever they afford [32] and also put on an apron/ outfit to cover wetting of their usual cloths to stay clean alike with women in Ebony state use [29]. Respondents avoided demanding activitiesand reduced fluid intake in an attempt to reduce the flow. On the other hand, in order to dilute the strong humiliating odor and irritating effect of urine, they were drinking more water; this impliesthat women were challenged in many ways and affected by feeding changes.

In order to save their marriage those living with their husband were trying to carry out their household activities, and maintain marital relationship satisfying husband’s sexual need though; they have no interest to do so; and also to protect the bed fromwetting during sexual act or the whole night the woman has to put protective clothes and change every time during the night before the husband sense wetness. Some respondents totally avoided association with people unless it is compulsory event such as funeral ceremony of a close relatives and religious gatherings where they sit at far end of the seat, mostly opt to sit down on theground and dark areas to hide the wetting if incase protection failed.

This study also displayed that people’s understanding on the condition and its perceived rareness made the women not to seek care and disclose their condition viewing as it happened only to them.Though they get information about the availability of treatment service after years of suffering, they experienced many challenges to reach the treatment center even after that, though few had supportive spouse or family who accompanied them to treatment center.

### Limitation

This study was conducted on women with obstetric fistula in hospital setting. Hence, experiences of women within communities, who either not aware of treatment availability or lacked support to access treatment might be different and not yet explored.

## Conclusion

The study showed that women had endured various social problems such as divorce, isolation and dependency. Beside they had difficulty of getting access to information and inability to afford products that can help manage their incontinence.They devised different strategies that might have both positive and negative health outcome to cope with the condition.

## Additional file

Additional file 1: Interview guide on social experiences and coping mechanisms of women with obstetric fistula. (DOCX 15 kb)

## Data Availability

The data sets used and/analysed during the current study are available from the corresponding authoron reasonable request.

## Abbreviations

EDHS: Ethiopian Demographic and Health Survey
OF: Obstetric Fistula
UNFPA: United Nations Population Fund
WAHAI: Women and Health Aliance International

## Footnotes

1 Pseudo name is used for confidentiality.
