# Supplementary material for: The loss of dignity: social experience and coping of women with obstetric fistula, in Northwest Ethiopia
Source: BMC Womens Health. 2019 Jul 1;19:84. doi: 10.1186/s12905-019-0781-7 (PMC6604173; doi:10.1186/s12905-019-0781-7)
Supplement: Supplementary file 1 — Interview guide on social experiences and coping mechanisms of women with obstetric fistula. (DOCX 15 kb) [file 12905_2019_781_MOESM1_ESM.docx]

**Additional file 1:** Interview guide on social experiences and coping mechanisms of women with obstetric fistula

| Introduction, consent and interview guides (probes used to ensure clarity) | | |
| --- | --- | --- |
| No. | Issues explored | Interview questions |
| 1 | Personal characteristics of the respondent | Could you tell me all about yourself?  (Your current age, age at fistula, address, marital status, education, no of children) |
| 2 | History of child birth circumstances | Would you tell be about your child birth experiences in delivery that caused this problem? What was the baby’s outcome?  How was fistula happened to you? |
| 3 | Patients understanding of condition | Tell me how did you recognize that you had fistula? |
| 4 | Experiences of women with obstetric fistula | Tell me your every day experience as a woman with this problem, regarding your relationship with your husband, family and neighbors.  What challenges did you faced while living with the problem?  What were you doing for your livelihood? |
| 5 | Ways to overcome challenges | How did you manage living with the leak? |
|  |  | What were you doing to overcome the social challenges? |
| 6 | Finding fistula treatment | How did you know that your problem is treatable?  How do you reach treatment center? |
